# Heterointerface Engineered Core-Shell Fe_2_O_3_@TiO_2_ for High-Performance Lithium-Ion Storage

**DOI:** 10.3390/molecules28196903

**Published:** 2023-10-01

**Authors:** Zeqing Miao, Kesheng Gao, Dazhi Li, Ziwei Gao, Wenxin Zhao, Zeyang Li, Wei Sun, Xiaoguang Wang, Haihang Zhang, Xinyu Wang, Changlong Sun, Yuanyuan Zhu, Zhenjiang Li

**Affiliations:** 1 Shandong Engineering Laboratory for Preparation and Application of High-Performance Carbon-Materials, College of Electromechanical Engineering, Qingdao University of Science and Technology, Qingdao 266061, China; 2 College of Materials Science and Engineering, Qingdao University of Science and Technology, Qingdao 266042, China; 3 Sino-German Institute of Technology, Qingdao University of Science and Technology, Qingdao 266100, China; 4 Key Laboratory of Spin Electron and Nanomaterials of Anhui Higher Education Institutes, Suzhou University, Suzhou 234000, China

**Keywords:** lithium-ion storage, iron-based anode, heterointerface engineering, built-in electric field, electrochemical kinetics

## Abstract

The rational design of the heterogeneous interfaces enables precise adjustment of the electronic structure and optimization of the kinetics for electron/ion migration in energy storage materials. In this work, the built-in electric field is introduced to the iron-based anode material (Fe_2_O_3_@TiO_2_) through the well-designed heterostructure. This model serves as an ideal platform for comprehending the atomic-level optimization of electron transfer in advanced lithium-ion batteries (LIBs). As a result, the core-shell Fe_2_O_3_@TiO_2_ delivers a remarkable discharge capacity of 1342 mAh g^−1^ and an extraordinary capacity retention of 82.7% at 0.1 A g^−1^ after 300 cycles. Fe_2_O_3_@TiO_2_ shows an excellent rate performance from 0.1 A g^−1^ to 4.0 A g^−1^. Further, the discharge capacity of Fe_2_O_3_@TiO_2_ reached 736 mAh g^−1^ at 1.0 A g^−1^ after 2000 cycles, and the corresponding capacity retention is 83.62%. The heterostructure forms a conventional p-n junction, successfully constructing the built-in electric field and lithium-ion reservoir. The kinetic analysis demonstrates that Fe_2_O_3_@TiO_2_ displays high pseudocapacitance behavior (77.8%) and fast lithium-ion reaction kinetics. The capability of heterointerface engineering to optimize electrochemical reaction kinetics offers novel insights for constructing high-performance iron-based anodes for LIBs.

## 1. Introduction

The increasing popularity of new-energy trams and the growing market demand for new-energy electronic devices have rendered electrochemistry and its related fields indispensable to the advancement of energy technology [1,2,3]. The advantages of no memory effect, extended lifespan, high energy density, and superior safety make lithium-ion batteries (LIBs) a prospective technology choice for future large-scale applications [4,5,6,7,8]. However, the limited theoretical capacity (372 mAh g^−1^) and inadequate rate performance of commercial graphite anodes impede the advancement of LIBs [9,10,11]. By identifying suitable anode materials to enhance capacity and kinetics, LIBs could offer superior energy/power density and broader application prospects. Transition metal oxides (TMOs), especially Fe_2_O_3_ with its high theoretical capacity (1007 mAh g^−1^) [12], abundance, strong chemical stability, and eco-friendliness, have attracted considerable interest [13,14,15,16,17,18]. However, the high lithiation potential, significant volume expansion, and side reaction tendencies diminish the feasibility of Fe_2_O_3_ as an LIB anode [19]. The use of frame support or core-shell structures to limit the volume expansion of Fe_2_O_3_ has proven to be an effective strategy. For example, Qi et al. synthesized Fe_2_O_3_/graphene nanocomposites by the high-pressure hydrothermal method, achieving a capacity of 1100.5 mAh g^−1^ at 0.2 A g^−1^ after 350 cycles [20]. Wang et al. developed C@Fe_2_O_3_/SWCNT nanocomposites, demonstrating favorable lithium-ion storage capacity with a reversible capacity of 1294.7 mAh g^−1^ at 0.05A g^−1^ [21]. He et al. prepared Fe_2_O_3_/C/rGO nanomaterials by the physical crosslinking method, attaining a reversible capacity of 609 mAh g^−1^ at 1.0 A g^−1^ while exhibiting excellent power density [22]. Although the strategy of composite or hybrid with carbon-based materials has proven effective in stabilizing electrochemical reactions and preventing physical crushing of Fe_2_O_3_ during lithiation/delithiation, some effective electron transfer is still sacrificed due to poor kinetics. Furthermore, the inadequate conductivity of Fe_2_O_3_ results in the significant polarization and degradation of lithium-ion storage performance at high current densities [23,24]. Therefore, the structure design and interface effect of Fe_2_O_3_ anodes are essential for high lithium-ion storage capacity.

Recognized for its remarkable structural stability, TiO_2_ has been dubbed a “zero-stress material” [25]. Compared to carbon-based materials, TiO_2_ possesses a higher lithium-ion inserting potential (1.5 V–1.8 V) and is less susceptible to the electroplating effect [25], thus TiO_2_ is often regarded as a safer choice for anode materials in LIBs. However, the low theoretical capacity of merely 167.5 mAh g^−1^ and inferior rate performance have impeded the research advancement of TiO_2_ [26]. Guan et al. designed S-doped TiO_2_@C nanosheets, achieving a capacity of 550 mAh g^−1^ at 0.3 C and a stable cycling life of 85.8% after 1000 cycles [27]. Hao et al. constructed O-deficient TiO_2_ with abundant active lithium-ion storage sites, offering a reversible capacity of 50 mAh g^−1^ at 100 C [28]. Xia et al. reported black TiO_2_ with a disordered surface could reach a remarkable reversible capacity of ~120 mAh g^−1^ at 0.04 C after 500 cycles [26]. Despite these advances, the sluggish lithium-ion diffusion kinetic remains a primary impediment constraining the lithium-ion storage capacity of TiO_2_. Constructing a heterostructure by coupling Fe_2_O_3_ and TiO_2_ represents an efficacious strategy for enhancing individual anode performance in lithium-ion storage. Fe_2_O_3_, an exemplary n-type semiconductor, and TiO_2_, a p-type semiconductor, form a heterostructure that triggers spontaneous electron movement towards the n-type semiconductor due to an electron concentration difference, leading to the creation of a built-in electric field at the heterointerface [29]. This built-in electric field can effectively improve the lithium-ion reaction kinetics, maintain long-term cycle stability, and maximize the synergistic effect of the heterostructure [30]. Therefore, the construction of a heterostructure signifies an optimal solution for augmenting the lithium-ion storage capacity of individual anodes (Fe_2_O_3_ or TiO_2_).

In this work, we propose feasible heterointerface engineering to fabricate a core-shell Fe_2_O_3_@TiO_2_ heterostructure as an advanced anode for LIBs through solvothermal and sol-gel progress. As expected, the well-designed Fe_2_O_3_@TiO_2_ heterostructure demonstrates remarkable lithium-ion storage performance, achieving an ultrahigh rate capacity, extraordinary cycling performance (1342 mAh g^−1^ at 0.1A g^−1^ after 300 cycles), and superior cycling durability (capacity retention of 83.6% at 1.0 A g^−1^ after 2000 cycles). The improvement of electrochemical kinetics for the Fe_2_O_3_@TiO_2_ heterostructure is confirmed by pseudocapacitance and electrochemical impedance spectroscopy (EIS). Our work fully exploits the synergistic effects of heterogeneous interfaces to enhance lithium-ion reaction kinetics and improve cycle durability, thereby providing valuable ideas for designing rational interface engineering and manufacturing advanced energy storage devices.

## 2. Results 

**Figure 1:**
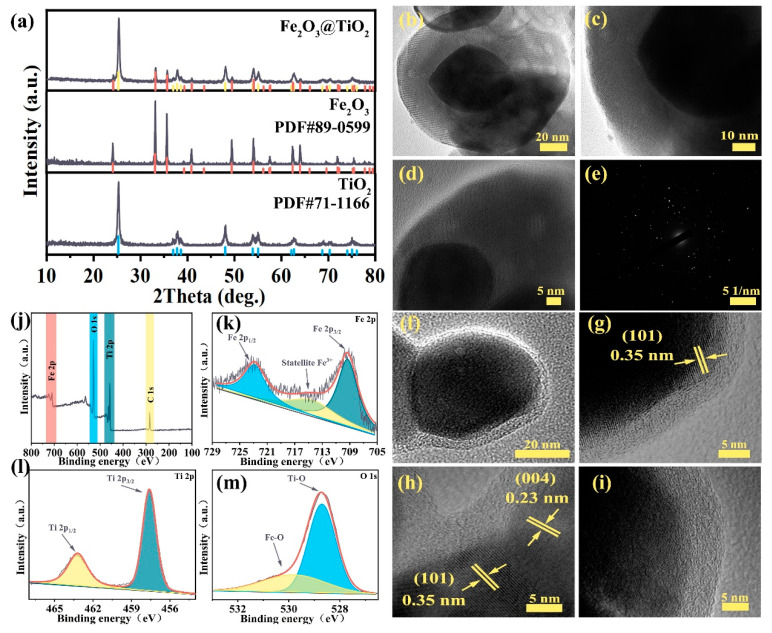
Characterization of Fe_2_O_3_@TiO_2_ heterostructure: (**a**) XRD patterns; (**b**–**i**) TEM, SAED, and HRTEM images; (**j**) XPS survey spectrum and high-resolution XPS survey spectra of (**k**) Fe 2p orbital, (**l**) Ti 2p orbital, and (**m**) O 1s orbital.

The detailed process route of the Fe_2_O_3_@TiO_2_ heterostructure is illustrated in Figure S1. The Fe_2_O_3_@TiO_2_ heterostructure is qualitatively examined by X-ray powder diffraction (XRD), initially. As shown in Figure 1a, the diffraction peaks located at 24.2°, 33.2°, 35.6°, 40.9°, 49.5°, 54.1°, 56.2°, 57.7°, 62.6°, and 64.1° can be indexed to (012), (104), (110), (113), (024), (116), (211), (018), (214), and (300) crystal planes of α-Fe_2_O_3_, respectively (PDF#89-0599). The diffraction peaks of the Fe_2_O_3_@TiO_2_ heterostructure and Fe_2_O_3_ particles exhibit negligible deviation from the standard cards, thereby confirming the successful synthesis of high-purity α-Fe_2_O_3_. Additionally, the diffraction peaks located at 25.3°, 36.9°, 37.9°, 38.6°, 48.1°, 55.1°, 62.7°, 68.8°, 70.4°, and 75.1° can correspond to (101), (103), (004), (112), (200), (211), (204), (220), and (215) crystal planes of anatase-TiO_2_ (PDF#71-1166), respectively. XRD results indicate that the Fe_2_O_3_@TiO_2_ heterostructure, Fe_2_O_3_ particles, and TiO_2_ particles have good crystallinity. Furthermore, the structure of the Fe_2_O_3_@TiO_2_ heterostructure is analyzed by transmission electron microscopy (TEM). The core-shell structure, as illustrated in Figure 1b, is distinguishable, with Fe_2_O_3_ particles completely wrapped by TiO_2_ (Figure 1c,d). Further, the selected area electron diffraction (SAED) of the Fe_2_O_3_@TiO_2_ heterostructure is shown in Figure 1e, revealing the Fe_2_O_3_@TiO_2_ heterostructure to be comprised of multiple nanocrystals and exhibiting excellent crystallinity, which aligns with the XRD results. A particle with a small shell thickness is selected to analyze the outer TiO_2_ shell, as shown in Figure 1f. The corresponding HRTEM image is presented in Figure 1g, exhibiting well-defined lattice fringes characteristic of the (101) crystal plane of anatase-TiO_2_, with a measured lattice spacing of 0.35 nm. In Figure 1h, distinct nanocrystals are observed, with the left lattice spacing of 0.35 nm corresponding to the (101) crystal plane of anatase-TiO_2_ and the right lattice spacing of 0.23 nm corresponding to the (004) crystal plane of anatase-TiO_2_, indicating that the outer shell comprises multiple anatase-TiO_2_ nanocrystals. In addition, a substantial abundance of disordered structures is observed within the outermost layer (Figure 1i), exhibiting similarities to previously reported black TiO_2_ [26]. The disordered surface facilitates the transition of Ti 2p orbital electrons to 3d orbitals, making it easier to capture charge carriers and improve the overall conductivity of TiO_2_ [31]. Furthermore, the presence of a significant quantity of disordered nanocrystals in TiO_2_ generates an intermediate energy state within the band gap, exhibiting a distinct energy distribution compared to the defect of crystals [32]. The contribution lies in the narrowing of the band-gap width and the enhancement of electrochemical kinetics [26]. Additionally, the elemental mapping results in Figure S2 reveal the homogeneous distribution of Fe, Ti, and O elements in Fe_2_O_3_@TiO_2_, whereby the Fe element is mainly provided by internal Fe_2_O_3_, Ti comes from the shell of TiO_2_, and O is derived from the Fe_2_O_3_, TiO_2_, and adsorbed oxygen. The surface element composition and valence state of Fe_2_O_3_@TiO_2_ heterostructure are analyzed by X-ray photoelectron spectroscopy (XPS). The presence of Ti, O, and Fe elements in Figure 1j can be determined based on the precise localization of characteristic peaks, without any indication of the existence of other impurity elements. The high-resolution XPS spectrum of the Fe 2p orbit is further presented in Figure 1k, the peak located at 722.9 eV corresponds to Fe 2p_1/2_, the peak located at 709.3 eV corresponds to Fe 2p_3/2_, and the small characteristic peak between Fe 2p_1/2_ and Fe 2p_3/2_ can be indexed to the satellite peak of Fe^3+^ [33]. The high-resolution XPS spectrum of the Ti 2p orbit is presented in Figure 1l. The peaks observed at 463.2 eV and 457.6 eV are attributed to the 2p_1/2_ and 2p_3/2_ orbitals of Ti, respectively [33]. Moreover, the high-resolution XPS spectrum of the O 1s orbit in Figure 1m exhibits well-defined peaks at 529.8 eV and 528.7 eV, corresponding to the Fe-O and Ti-O bonds, respectively [33]. These XPS results provide compelling evidence for the successful synthesis of both Fe_2_O_3_ and TiO_2_.

**Figure 2:**
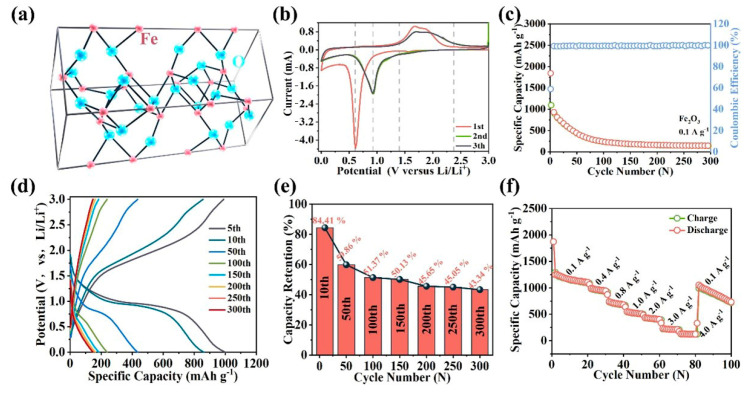
(**a**) Crystal structure image of α-Fe_2_O_3_. Electrochemical properties of Fe_2_O_3_ as anode material for LIBs: (**b**) CV curves at a scan rate of 0.1 mV s^−1^; (**c**) cycling performance at a current density of 0.1 A g^−1^, (**d**) GCD curves, (**e**) capacity retention and linear fit curve; (**f**) rate performance from 0.1 A g^−1^ to 4.0 A g^−1^.

The crystal structure of α-Fe_2_O_3_, corresponding to a rhomboidal crystal system, is depicted in Figure 2a. The standard half-cell test procedure is employed to conduct the testing of Fe_2_O_3_. To elucidate the underlying mechanism of lithium-ion storage, an initial cyclic voltammetry (CV) test is performed (Figure 2b). The significant reduction reaction occurs at 0.61 V during the initial discharge process, which can be interpreted as the formation mechanism of the solid electrolyte interphase (SEI) film [34]. During the initial charge process, a distinct oxidation peak is observed between 1.4 V to 2.26 V, indicating a continuous Fe^0^ to Fe^3+^ oxidation process, consistent with previous reports [34]. The CV curves of the second and third cycles exhibit a remarkable alteration compared to that of the first cycle. During the discharge process, a novel reduction peak emerges at 0.92 V, indicating an electrochemical reaction described by Equation (1) [35].

(1) $${Fe}_{2}O_{3}+{6Li}^{+}+{6e}^{-}\to {2Fe}^{0}+3{Li}_{2}O$$

Additionally, compared to the charging process of the initial cycle, a discernible shift toward higher voltage is observed in the oxidation. This shift may be attributed to a polarization phenomenon caused by surface-insulating lithium compounds [36]. The half-cell is tested at 0.1 A g^−1^ for the cycle performance (Figure 2c). The discharge capacity reaches 1844.6 mAh g^−1^, while the charge capacity amounts to 1095.1 mAh g^−1^ in the first cycle, resulting in an initial Coulombic efficiency (ICE) of 59%. The ultra-high ICE indicates a substantial conversion of lithium-ion into irreversible Li-containing compounds during the initial charge/discharge process [37]. Indeed, the capacity exhibits a declining trend over 300 cycles, with the capacity dropping to only 146.8 mAh g^−1^ at the 300th cycle. To explore the reason for the capacity decrease, the GCD curves are analyzed in Figure 2d. The voltage platform exhibits a pronounced decline and eventually diminishes during the repeated charge/discharge process, indicating the deactivation of the initial electrochemical reaction. The capacity retention exhibits a significant decline in Figure 2e, with a mere 14.66% remaining at the 300th cycle, indicating the gradual failure of Fe^3+^ and a corresponding decrease in effective electron transfer. The results of the capacity retention are consistent with the GCD curves. Furthermore, the rate performance of the Fe_2_O_3_ anode is examined in Figure 2f, ranging from 0.1 A g^−1^ to 4.0 A g^−1^. Remarkably, at the 20th cycle, the discharge capacity reaches an impressive value of 1108 mAh g^−1^. Upon reaching a current density of 0.1 A g^−1^, the discharge capacity reverts to 1049 mAh g^−1^, leading to a significant decrease. Ultimately, the discharge capacity only reaches 731 mAh g^−1^ after the 100th cycle, indicating a suboptimal rate capacity of Fe_2_O_3_. Moreover, the Fe_2_O_3_ anode is tested in a long-term cycle at 1.0 Ag^−1^, as shown in Figure S3a. The initial discharge capacity stands at 1479.5 mAh g^−1^, while the first charge capacity reaches 981.5 mAh g^−1^, with an ICE of 66%. The discharge capacity exhibits a continuous decline with repeated charge/discharge, ultimately reaching only 99 mAh g^−1^ after 2000 cycles. The capacity retention depicted in Figure S3b accurately represents the electrochemical behavior of the Fe_2_O_3_ anode. The capacity retention gradually decreases to 32% until the 35th cycle, followed by a relatively stable retention from the 35th cycle to the 1200th cycle. Eventually, the capacity retention drops to 12.23% at the 2000th cycle. Combined with the electrochemical test results, the Fe_2_O_3_ anode can deliver extremely high capacity, but it cannot be maintained. The primary cause lies in the gradual deactivation of Fe^3+^ during repeated charge/discharge cycles, which arises from the pronounced volume expansion during repeated lithiation/delithiation.

**Figure 3:**
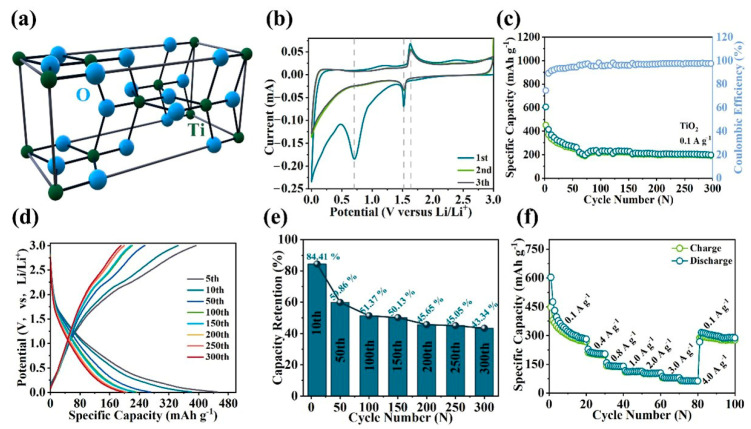
(**a**) Crystal structure image of anatase-TiO_2_. Electrochemical properties of TiO_2_ as anode material for LIBs: (**b**) CV curves at a scan rate of 0.1 mV s^−1^; (**c**) cycling performance at a current density of 0.1 A g^−1^, (**d**) GCD curves, (**e**) capacity retention and fitted linear curve; (**f**) rate performance from 0.1 A g^−1^ to 4.0 A g^−1^.

The crystal structure of anatase-TiO_2_ is shown in Figure 3a. The electrochemical performances of the TiO_2_ anode are analyzed by a standard half-cell test process. Figure 3b shows the CV curves of TiO_2_ anode at a scan rate of 0.1 mV s^−1^. The reduction peak observed at 1.52 V in the initial discharge process can be regarded as the lithium-ion insertion, as exemplified by Equation (2) [28].

(2) $${TiO}_{2}+{xLi}^{+}{xe}^{-}\to {Li}_{x}{TiO}_{2}$$

The emergence of the second reduction peak at 0.7 V can be attributed to the formation of the SEI film on the surface [38]. During the charge process, the oxidation peak located at 1.6 V corresponds to the process of lithium-ion desertion [38]. The CV curves of the second and third cycles exhibit a remarkable fit, thereby indicating the inherent stability of the electrochemical reaction. Figure 3c shows the cycling performance of the TiO_2_ anode at 0.1 A g^−1^. In the first charge/discharge process, the discharge capacity reaches 605.3 mAh g^−1^, the charge capacity reaches 450.7 mAh g^−1^, and the corresponding ICE is 74%. The exceptional discharge capacity can be ascribed to the disordered surface, offering an abundance of active lithium-ion storage sites [26]. The discharge capacity exhibits a continuous decline from the initial cycle to the 50th cycle, which could be related to the activation process of TiO_2_. The discharge capacity exhibits minimal fluctuations over the subsequent 250 cycles and eventually maintains 205 mAh g^−1^ at the 300th cycle. The extraordinary cycle durability can be attributed to the enhanced structural adaptability of the disordered surface during charge/discharge processes [26]. Further, the GCD curves of TiO_2_ are shown in Figure 3d. The excellent degree of curve fitting indicates minimal polarization. The electrochemical stability of TiO_2_ is demonstrated by the maintenance of a distinct voltage plateau over more than 300 cycles. Figure 3e shows the capacity retention at 0.1 A g^−1^, which remains stable at 50% after 100 cycles. The rate performance of the TiO_2_ anode is shown in Figure 3f. The discharge capacity reaches 281 mAh g^−1^ at the 20th cycle at 0.1 A g^−1^. Upon restoring the current density to 0.1 A g^−1^, the discharge capacity returns to 315 mAh g^−1^ and exhibits sustained stability. The stability demonstrated by the rate capacity is consistent with the capacity retention, which can be attributed to the reduction in the energy barrier of lithium-ion transport in the disordered surface of the TiO_2_ [39]. Moreover, the long-term cycle test is shown in Figure S4a. The discharge capacity of 172.3 mAh g^−1^ can still be achieved after 2000 cycles at 1.0 A g^−1^, and the Coulomb efficiency is always maintained at ~100%. In Figure S4b, the cycle durability of TiO_2_ is further observed by calculating the capacity retention. The capacity retention of the 10th cycle is 83.63%. The capacity retention exhibits a slight decline from the 10th to the 200th cycle, followed by an increase to approximately 85%. Finally, the capacity retention is still maintained at 82.63% in the 2000th cycle. In conjunction with the findings from the aforementioned electrochemical tests, the TiO_2_ anode exhibits exceptional cycling durability, a stable electrochemical reaction process, and minimal polarization phenomenon, thereby effectively mitigating volume expansion and enhancing electrical conductivity.

**Figure 4:**
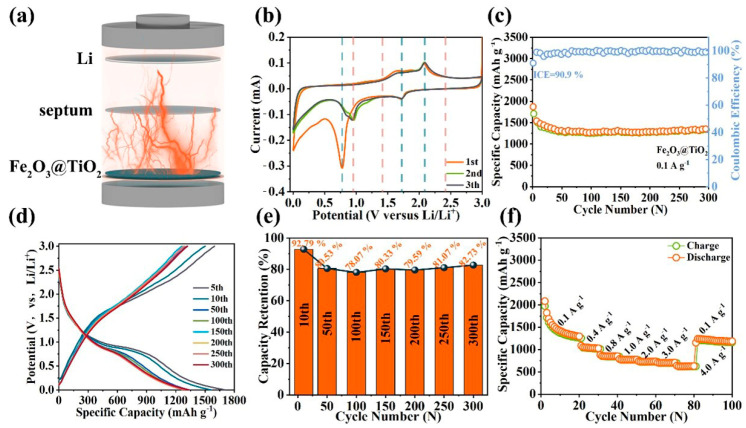
(**a**) Model image of half-cell. Electrochemical properties of Fe_2_O_3_@TiO_2_ heterostructure as anode material for LIBs: (**b**) CV curves at a scan rate of 0.1 mV s^−1^; (**c**) cycling performance at a current density of 0.1 A g^−1^, (**d**) GCD curves, (**e**) capacity retention and fitted linear curve; (**f**) rate performance from 0.1 A g^−1^ to 4.0 A g^−1^.

The working Image of the half-cell with the Fe_2_O_3_@TiO_2_ heterostructure as the anode is shown in Figure 4a. Firstly, the electrochemical reactions of the Fe_2_O_3_@TiO_2_ heterostructure are explored by the CV test, with the CV curves from the first to third cycles shown in Figure 4b. During the initial discharge process, a prominent reduction peak at 1.72 V indicates the lithium-ion insertion into the TiO_2_ shell, as described by Equation (2). Further, the second reduction peak at 0.77 V corresponds to the SEI film generation of TiO_2_, echoing the reduction peak of SEI film generation in Figure 3b. During the first oxidation process, the oxidation peak ranging from 1.41 V to 2.42 V corresponds to the continuous transformation of Fe^0^ to Fe^3+^. In addition, the oxidation peak located at 2.1 V indicates the lithium-ion deinsertion from TiO_2_, which is consistent with previous results. The positions of the oxidation/reduction peaks of the second cycle remain unchanged, indicating the excellent electrical conductivity of the Fe_2_O_3_@TiO_2_ heterostructure. The emergence of a new reduction peak at 0.92V in the second and third CV curves, a process shown in Equation (1), further substantiates the lithiation of Fe_2_O_3_. The broader reduction peak of Fe_2_O_3_ in the third CV curve compared to the second suggests a progressive and complete lithiation of Fe_2_O_3_. The CV test results imply that the Fe_2_O_3_@TiO_2_ heterostructure engages in both lithium-ion insertion/deinsertion and reduction/oxidation, thereby demonstrating superior electrochemical stability. Figure 4c shows the short-term cycle performance of the Fe_2_O_3_@TiO_2_ heterostructure at 0.1 A g^−1^. During the initial insertion/desertion process, the discharge and charge capacities are recorded as 1869 mAh g^−1^ and 1697 mAh g^−1^, respectively, resulting in an impressive ICE of 90.9%. The high discharge capacity is ascribed to the abundance of active lithium-ion storage sites on the disordered surface of TiO_2_ and the enhanced charge transfer characteristics of Fe_2_O_3_. The high Coulomb efficiency is attributed to the synergistic effect after hybridization, which enhances the electrochemical reaction stability. The cycle curve reveals a gradual decrease in discharge capacity from the 1st cycle to the 50th cycle. Subsequently, the Fe_2_O_3_@TiO_2_ heterostructure exhibits fluctuating discharge capacity around 1300 mAh g^−1^ after 30 cycles, ultimately reaching 1342 mAh g^−1^ at the 300th cycle. Furthermore, the electrochemical process is further explored through the GCD curves in Figure 4d. The GCD curve of the fifth cycle fits well with the 10th cycle and exhibits a large discharge platform from 1 V to 0.6 V, implying excellent electrical conductivity and structural stability of the Fe_2_O_3_@TiO_2_ heterostructure. When compared with the GCD curves of Fe_2_O_3_, the Fe_2_O_3_@TiO_2_ heterostructure shows better electrochemical stability and maintains a highly reversible electrochemical reaction during 300 cycles. The cycle durability of Fe_2_O_3_@TiO_2_ heterostructure is verified by the capacity retention in Figure 4e. Compared with the first cycle, the capacity retention can exhibit 92.79% at 10 cycles, and it is stable at approximately 80% after the 50th cycle. Comparing the capacity retention of Fe_2_O_3_ and TiO_2_, it can be observed that the capacity retention of the Fe_2_O_3_@TiO_2_ heterostructure is significantly higher than both individual anodes due to their synergistic effect. Moreover, the TiO_2_ shell effectively alleviates the volume expansion and successfully maintains the electrochemical activity of Fe_2_O_3_. The rate performance of the Fe_2_O_3_@TiO_2_ heterostructure is exhibited in Figure 4f. The discharge capacity of the Fe_2_O_3_@TiO_2_ heterostructure is 1297 mAh g^−1^ after 20 cycles at 0.1 A g^−1^. Upon restoring the current density to 0.1 A g^−1^, the discharge capacity can be reinstated to 1217 mAh g^−1^. This result suggests that the built-in electric field can improve the lithium-ion reaction kinetics and the heterogeneous interface can provide more active sites for lithium-ion insertion. Figure S5a shows the cycle curve of the Fe_2_O_3_@TiO_2_ heterostructure during 2000 cycles at 1.0 A g^−1^. The initial cycle exhibits a remarkable discharge capacity of 1555 mAh g^−1^ and an impressive charge capacity of 1274 mAh g^−1^ for the Fe_2_O_3_@TiO_2_ heterostructure. Compared with Fe_2_O_3_ and TiO_2_, the ICE of the Fe_2_O_3_@TiO_2_ heterostructure shows a great improvement of 82%. The enhanced electrochemical stability can be attributed to the protective effect of the core-shell structure, which effectively mitigates volume expansion. The discharge capacity reaches 736 mAh g^−1^, while maintaining a consistent Coulomb efficiency of ~100% even after the 2000th cycle at 1.0 A g^−1^. Compared with the long-term cycle properties shown in Figure 2a and Figure 3, Fe_2_O_3_@TiO_2_ heterostructure exhibits better capacity and more stable Coulomb efficiency. The capacity retention of the Fe_2_O_3_@TiO_2_ heterostructure is calculated in Figure S5b. Compared to the first discharge capacity, the capacity retention of the 10th cycle is 80.73%, exhibiting a slight decrease in capacity retention before the 150th cycle. It is worth noting that between the 200th cycle and the 1600th cycle, the capacity retention rises and stabilizes at around 95%. Eventually, the capacity retention can still be maintained at 83.62% in the 2000th cycle. In summary, after a series of electrochemical tests, the Fe_2_O_3_@TiO_2_ heterostructure has better lithium-ion storage capacity than Fe_2_O_3_ and TiO_2_. Further, in order to comprehensively evaluate the lithium-ion storage performance of the Fe_2_O_3_@TiO_2_ heterostructure, both the discharge capacity and cycle life for the iron-based anodes are collected in Table S1. Obviously, the Fe_2_O_3_@TiO_2_ heterostructure achieves superior discharge capacity and cycle life compared to other reported iron-based anodes, indicating its extensive application potential as an anode for LIBs. The higher discharge capacity is provided by the synergistic effect of the heterogeneous interface and abundant active lithium-ion storage sites. The smaller polarization phenomenon could be related to the improved electron/ion migration kinetics of the built-in electric field. The enhanced durability of the longer cycle contributes to effectively mitigating the volume expansion during repeated lithium-ion insertion/desertion, thereby showing the remarkable efficacy of the TiO_2_ shell.

**Figure 5:**
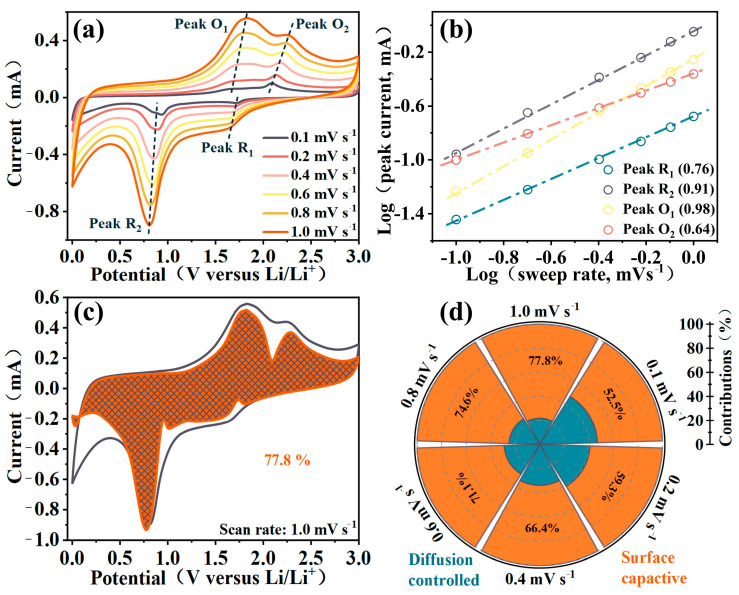
(**a**) CV curves of Fe_2_O_3_@TiO_2_ heterostructure at different scan rates from 0.1 to 1.0 mV s^−1^; (**b**) the *b*-values of corresponding oxidation peaks and reduction peaks; (**c**) area ratio image of pseudocapacitance at 1.0 mV s^−1^; (**d**) ratio image of pseudocapacitance contribution at different scan rates from 0.1 to 1.0 mV s^−1^.

The pseudocapacitance behavior of the Fe_2_O_3_@TiO_2_ heterostructure is investigated through CV testing at various scan rates. The CV curves of the Fe_2_O_3_@TiO_2_ heterostructure at different scan rates are shown in Figure 5a. The observed trend suggests that, as the scan rate increases, the shape of the CV curves remains consistent. This observation provides strong evidence for high electrochemical stability and minimal polarization [40], which is consistent with the result of rate capacity.

(3) $$\;i={av}^{b}$$

The *b*-value can be determined by the relationship between the current (*i*) and the scan rate (*v*), as depicted in Equation (3). *a* is a constant, thus the *b*-value can be calculated from the slope of log(*i*) and log(*v*) [41]. The *b*-value is a critical parameter for evaluating diffusion-controlled and surface capacitive-controlled processes. When the *b*-value is less than 0.5, the electrochemical reaction is diffusion progress; and when the *b*-value is higher than 1, the electrochemical reaction is completely capacitive behaviors [42]. For the oxidation peaks and reduction peaks belonging to the Fe_2_O_3_@TiO_2_ heterostructure, *b*-values are calculated and linearly fitted, and the results are shown in Figure 5b. All *b*-values are between 0.5 and 1, indicating that pseudocapacitance behavior exists in the electrochemical process of the Fe_2_O_3_@TiO_2_ heterostructure.

(4) $$i=k_{1}v+k_{2}v^{1/2}$$

The behavior of pseudocapacitance can be quantitatively analyzed according to Equation (4) [43], where *i* is the current, and *v* is the scan rate. At a particular voltage, *k*_1_ and *k*_2_ can be treated as constants. According to the calculated results, Figure 5c shows the pseudocapacitance behavior ratio at 1.0 mV s^−1^. In fact, the results demonstrate the proportion of pseudocapacitance behavior is as high as 77.8%, and high pseudocapacitance behavior can improve the lithium-ion reaction kinetics, which is consistent with the previous electrochemical analysis. Further, according to Figure 5d, pseudocapacitance behavior dominates the electrochemical process of Fe_2_O_3_@TiO_2_ heterostructure. In addition, EIS analysis is further performed to investigate the electrical conductivity, shown in Figure S6. The fitted charge-transfer resistance of Fe_2_O_3_@TiO_2_ (99.3 Ω) is much smaller than that of Fe_2_O_3_ (342.1 Ω) or TiO_2_ (160.8 Ω), and the slope of Fe_2_O_3_@TiO_2_ in the low-frequency region is larger, showing higher electronic conductivity. The reason for the high pseudocapacitance behavior and high electronic conductivity can be attributed to the disordered surface providing more active lithium-ion storage sites and the built-in electric field bringing more powerful electron/ion mobility.

## 3. Conclusions

In conclusion, the core-shell Fe_2_O_3_@TiO_2_ heterostructure has been successfully prepared through heterointerface engineering. The well-designed heterogeneous interface between Fe_2_O_3_ and TiO_2_ forms conventional p-n junctions, forming a built-in electric field that enhances electrochemical kinetics. The meticulously engineered core-shell structure effectively mitigates volumetric expansion and imparts exceptional cycling durability to the Fe_2_O_3_@TiO_2_ heterostructure (capacity retention of 83.62% after 2000 cycles). The disordered surface provides abundant active sites for lithium-ion storage (discharge capacity of 1342 mAh g^−1^ at 0.1 A g^−1^), reducing the lithium-ion diffusion barrier and increasing the capacity. This study underscores the pivotal role of designing semiconducting heterostructures in enhancing electrochemical activity, thereby paving the way for novel applications of heterogeneous electrode materials.

## Data Availability

The data that support the findings of this study are available from the corresponding author upon reasonable request.

## Supplementary Materials

The following supporting information can be downloaded at: https://www.mdpi.com/article/10.3390/molecules28196903/s1. Refs. [44,45,46,47,48,49,50,51,52] are cited in Supplementary Materials.

## Sample Availability

Samples of the compounds are available from the authors.
