# Supplementary material for: Heterointerface Engineered Core-Shell Fe2O3@TiO2 for High-Performance Lithium-Ion Storage
Source: Molecules. 2023 Oct 1;28(19):6903. doi: 10.3390/molecules28196903 (PMC10574312; doi:10.3390/molecules28196903)
Supplement: Supplementary file 1 [file molecules-28-06903-s001.zip › molecules-2625963-supplementary.pdf]

Heterointerface Engineered Core-Shell Fe_2O_3@TiO_2 for High-Performance Lithium-Ion Storage

Contents:

S1. Characterization methods and details analysis

S2. Results and discussion

Figure S1. Preparation of Fe_2O_3@TiO_2 heterostructure.

Figure S2. TEM image and the corresponding elemental mapping of Fe, Ti, O for Fe_2O_3@TiO_2 heterostructure.

Figure S3. Electrochemical properties of Fe_2O_3 as anode material for LIBs.

Figure S4. Electrochemical properties of TiO_2 as anode material for LIBs.

Figure S5. Electrochemical properties of Fe_2O_3@TiO_2 as anode material for LIBs.

Figure S6. EIS image of Fe_2O_3, TiO_2 and Fe_2O_3@TiO_2.

Table S1. Table of electrochemical performance comparison.

Reference

S1. Characterization methods and details analysis

S1.1. Materials characterization

Transmission electron microscopy (TEM, Tecnai 20U-Twin microscope) is used to examine the morphology. X-ray diffraction (XRD, X'pert Pro MPD) patterns are measured on a diffractometer with Cu-Kα radiation (λ = 0.15443 nm). Raman spectra are collected with 532 nm photons from an Ar^+ laser. X-ray photoelectron spectroscopy (XPS) is inspected on the Thermo ESCALAB 250.3.

S1.2. Device assembly measurements

The CR2016-type coin cells are assembled in an argon-filled glove box. Li metal is used as the counter and reference electrode. The positive and negative electrodes are electronically separated by a polypropylene film (Celgard 2320) saturated with electrolytes. The electrolyte solution is $LiPF_6$ (1 M) in ethylene carbonate/dimethyl carbonate/diethyl carbonate (1:1:1 vol %). The cycling and rate performances are recorded on a NEWARE battery measurement system, and cyclic voltammetry (CV) is performed using a CHI660D electrochemical workstation (Shanghai CH Instruments Co., China). Electrochemical impedance spectroscopy (EIS) measurements are also carried out on the CHI660D electrochemical workstation.

S2. Results and discussion

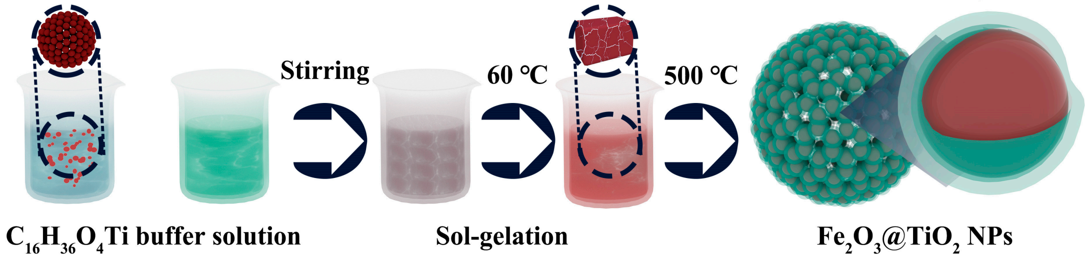

Figure S1. Preparation of Fe_2O_3@TiO_2 heterostructure.

Fe_2O_3@TiO_2 preparation is divided into three steps. As shown in Figure S1, Fe_2O_3 particles are prepared by hydrothermal method. The specific scheme is to dissolve 1 g $FeCl_3 \cdot 6H_2O$ into 30 ml deionized water, and stirring continuously. Then 0.5 g polyvinylpyrrolidone (PVP) is added into the solution, continuously stirring. The solution is transferred into a polytetrafluoroethylene lining and assembled into a high-pressure reactor. Fe_2O_3 particles are obtained by holding at 200 °C for 600 min. The second step is the sol-gel process. Firstly, the buffer solution is prepared, and the 20 ml C_2H_6O is mixed with 2 ml CH_3COOH. After evenly mixing, 5 ml of $C_{16}H_{36}O_4Ti$ is slowly added and continuously stirred. The pre-prepared Fe_2O_3 particles are ultrasonically dispersed into 3 ml deionized water, and 10 ml anhydrous ethanol is added to stir well, then the pH value is adjusted with HCl to 4, referred to as dispersion solution 1. Add dispersion solution 1 to the buffer solution slowly, stirring continuously until a gel forms. The gel is aged for 24 h and then put into a vacuum drying box at 60 °C for drying. The powder obtained after drying is fully ground with a mortar. Then it was put into a tube furnace and heated at a heating rate of 10 °C min^{-1} to 500 °C under argon atmosphere for 30 min. After natural cooling, reddish-brown powder is prepared as Fe_2O_3@TiO_2.

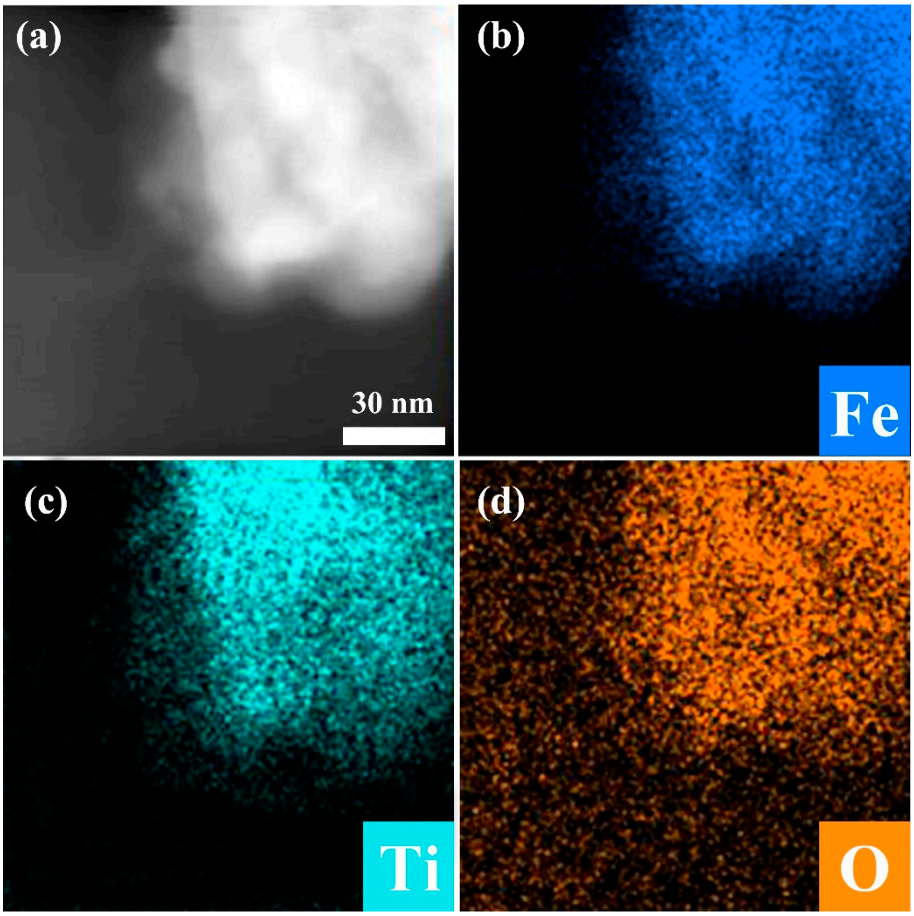

Figure S2. (a) TEM image and (b-d) the corresponding elemental mapping of Fe, Ti, O for Fe_2O_3@TiO_2 heterostructure.

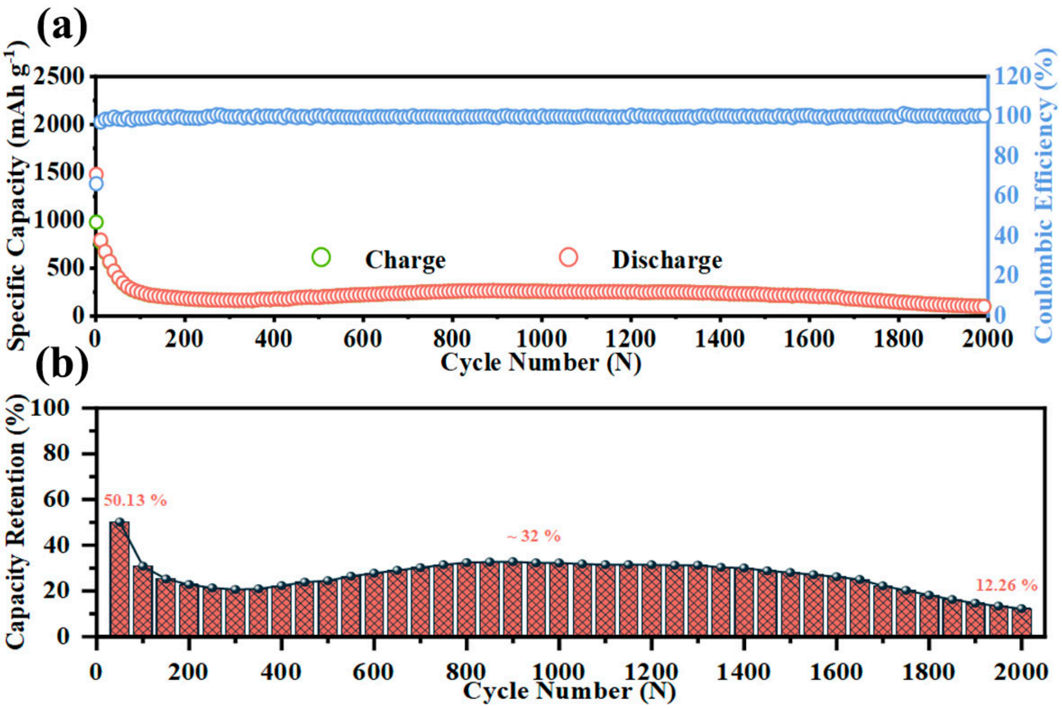

Figure S3. Electrochemical properties of Fe_2O_3 as anode material for LIBs: (a) cycling performance at a current density of 1.0 A g^{-1}, (b) corresponding capacity retention and fitted linear curve.

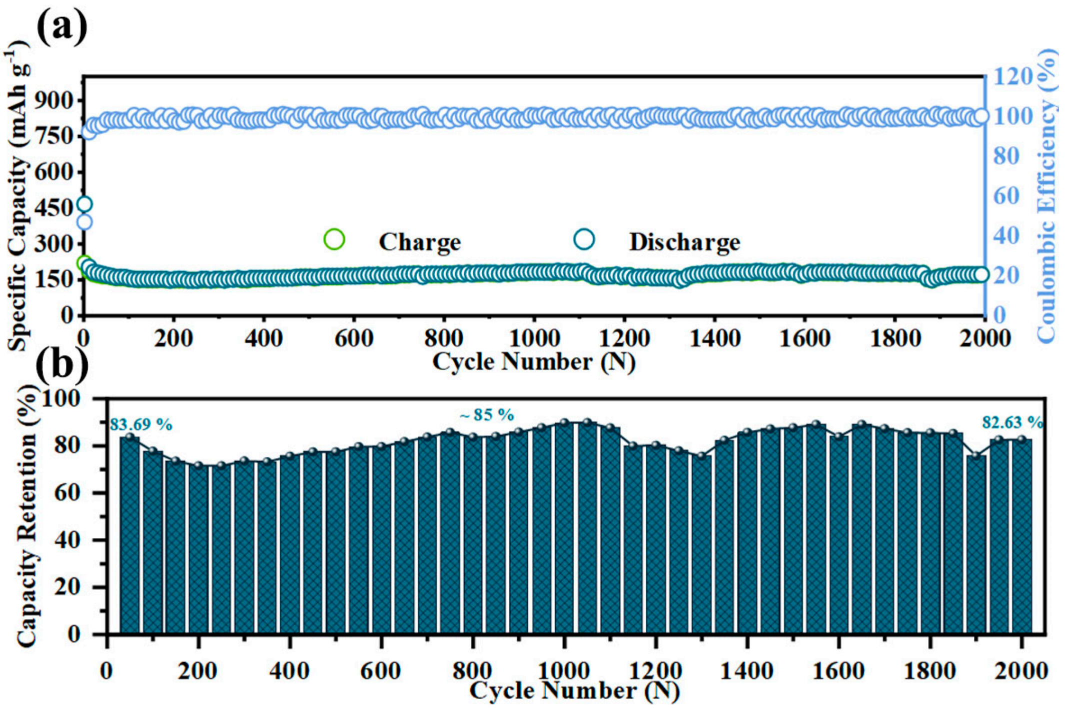

Figure S4. Electrochemical properties of TiO_2 as anode material for LIBs: (a) cycling performance at a current density of 1.0 A g^{-1}, (b) corresponding capacity retention and fitted linear curve.

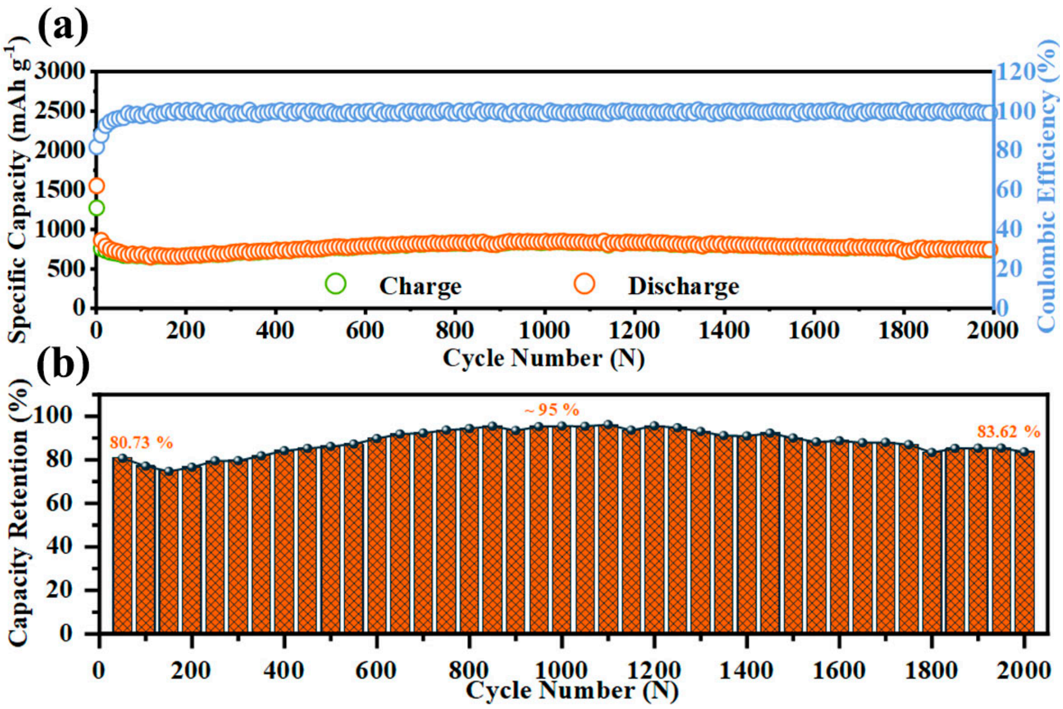

Figure S5. Electrochemical properties of Fe_2O_3@TiO_2 as anode material for LIBs: (a) cycling performance at a current density of 1.0 A g^{-1}, (b) corresponding capacity retention and fitted linear curve.

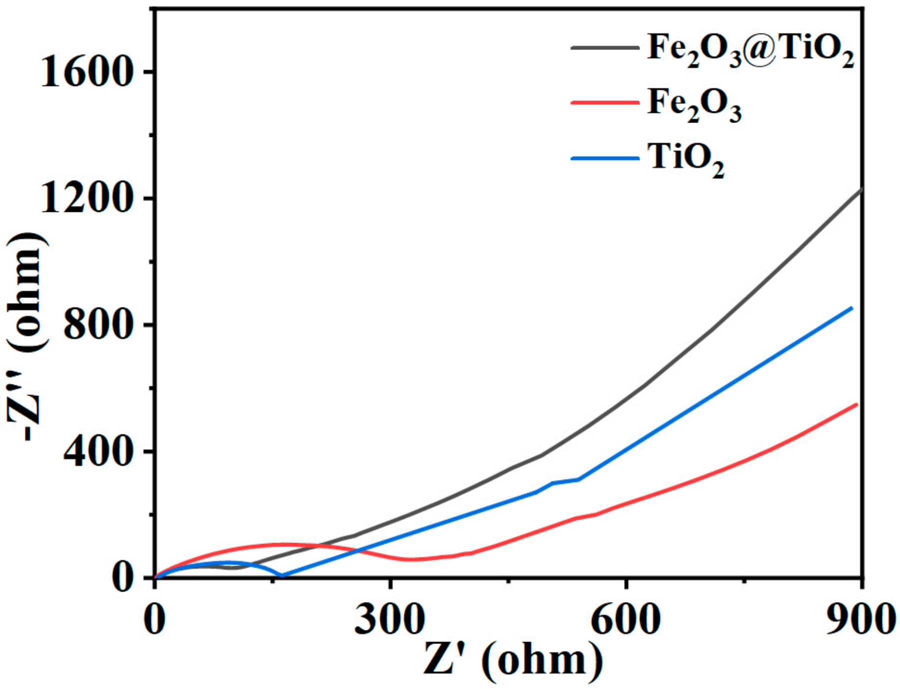

Figure S6. EIS image of Fe_2O_3, TiO_2 and Fe_2O_3@TiO_2.

Table S1. Table of electrochemical performance comparison.

Material	Current density	Cycle number	Discharge capacity	Ref.
This work	0.1 A g^{-1}	300	1342 mAh g^{-1}	/
Fe_3O_4@C	0.5 C	200	~1200 mAh g^{-1}	[1]
Fe_2O_3/rGO	0.2 A g^{-1}	30	704 mAh g^{-1}	[2]
Ti-Fe_2O_3@rGO	0.2 A g^{-1}	120	1155 mAh g^{-1}	[3]
TiO_2@Fe_2O_3	0.12 A g^{-1}	150	497 mAh g^{-1}	[4]
Fe_2O_3@TiO_2 nanotube	0.2 A g^{-1}	150	450 mAh g^{-1}	[5]
Fe_2O_3@$Li_4Ti_5O_{12}$	0.1 A g^{-1}	30	~300 mAh g^{-1}	[6]
Fe_3O_4@C	0.05 A g^{-1}	40	1080 mAh g^{-1}	[7]
Fe_2N@amorphous carbon	0.5 A g^{-1}	500	534 mAh g^{-1}	[8]
Fe_2O_3/Fe_3O_4@Carbon	0.5 A g^{-1}	200	1210 mAh g^{-1}	[9]
C@Fe_2O_3/SWCNT	0.05 A g^{-1}	200	1294 mAh g^{-1}	[10]

References

1. Liu, Z.; Yu, X.-Y.; Paik, U., Etching-in-a-Box: A Novel Strategy to Synthesize Unique Yolk-Shelled Fe_3O_4@Carbon with an Ultralong Cycling Life for Lithium Storage. *Adv. Energy Mater.* **2016,** 6, (6), 1502318.
2. Wang, L.; Chen, Q.; Zhu, Y.; Qian, Y., Graphene-wrapped Fe_2O_3 nanorings for Li ion battery anodes. *Chin. Sci. Bull.* **2014,** 59, (32), 4271-4273.
3. Lin, Y.; Sun, L.; Hu, J.; Tan, H.; Xie, F.; Qu, Y.; Wang, K.; Zhang, Y., Ti-doped Fe_2O_3/carbon cloth anode with oxygen vacancies and partial rGO encapsulation for flexible lithium ion batteries. *J. Alloys Compd.* **2022,** 924, 166441.
4. Luo, Y.; Luo, J.; Jiang, J.; Zhou, W.; Yang, H.; Qi, X.; Zhang, H.; Fan, H. J.; Yu, D. Y. W.; Li, C. M.; Yu, T., Seed-assisted synthesis of highly ordered TiO_2@α-Fe_2O_3 core/shell arrays on carbon textiles for lithium-ion battery applications. *Energy Environ. Sci.* **2012,** 5, (4), 6559-6566.
5. Lv, X.; Deng, J.; Sun, X., Cumulative effect of Fe_2O_3 on TiO_2 nanotubes via atomic layer deposition with enhanced lithium ion storage performance. *Appl. Surf. Sci.* **2016,** 369, 314-319.
6. Chen, M.; Li, W.; Shen, X.; Diao, G., Fabrication of Core–Shell α-Fe_2O_3@ $Li_4Ti_5O_{12}$ Composite and Its Application in the Lithium Ion Batteries. *ACS Appl. Mater. Interfaces* **2014,** 6, (6), 4514-4523.
7. Su, L.; Zhong, Y.; Zhou, Z., Role of Transition Metal Nanoparticles in the Extra Lithium Storage Capacity of Transition Metal Oxides: A Case Study of Hierarchical Core–shell Fe_3O_4@C and Fe@C Microspheres. *J. Mater. Chem. A* **2013,** 1, (47), 15158-15166.
8. Ding, R.; Zhang, J.; Zhang, J.; Li, Z.; Wang, C.; Chen, M., Core-shell Fe_2N@amorphous carbon nanocomposite-filled 3D graphene framework: An additive-free anode material for lithium-ion batteries. *Chem. Eng. J.* **2019,** 360, 1063-1070.
9. Li, Y.; Fu, Y.; Chen, S.; Huang, Z.; Wang, L.; Song, Y., Porous Fe_2O_3/Fe_3O_4@Carbon octahedron arrayed on three-dimensional graphene foam

for lithium-ion battery. *Composites, Part B* **2019,** 171, 130-137.

10. Wang, W.; Feng, Y.; Zhang, S.; Wang, M.; Song, W.; Yue, L.; Ge, M.; Mi, J., Facile premixed flame synthesis C@Fe_2O_3/SWCNT as superior free-standing anode for lithium-ion batteries. *J. Alloys Compd.* **2022,** 905, 164247.
